# The development and characterization of an *E. coli* O25B bioconjugate vaccine

**DOI:** 10.1007/s10719-021-09985-9

**Published:** 2021-03-17

**Authors:** Michael Kowarik, Michael Wetter, Micha A. Haeuptle, Martin Braun, Michael Steffen, Stefan Kemmler, Neil Ravenscroft, Gianluigi De Benedetto, Matthias Zuppiger, Dominique Sirena, Paola Cescutti, Michael Wacker

**Affiliations:** 1 GlycoVaxyn AG, Grabenstrasse 3, 8952 Schlieren, Switzerland; 2 LimmaTech Biologics AG, Grabenstrasse 3, 8952 Schlieren, Switzerland; 3 Present Address: Institute of Microbiology, ETH Zurich, Vladimir-Prelog-Weg 1-5/10, 8093 Zürich, Switzerland; 4 Present Address: Molecular Partners AG, Wagistrasse 14, 8952 Schlieren, Switzerland; 5 Present Address: Numab Therapeutics AG, Einsiedlerstrasse 34, 8820 Wädenswil, Switzerland; 6 Department of Chemistry, University of Cape Town, Rondebosch, 7701 South Africa; 7 Dip. di Scienze della Vita, University di Trieste, 34127 Trieste, Italy; 8 Present Address: National Institute for Biological Standards and Control, Blanche Lane, South Mimms, Potters Bar, Hertfordshire, EN6 3QG UK; 9 Present Address: GlycoEra AG, Grabenstrasse 3, 8952 Schlieren, Switzerland; 10 Present Address: Wacker Biotech Consulting AG, Heuelstrasse 22, 8800 Thalwil, Switzerland

**Keywords:** *Escherichia coli*, Serotype O25B, Bioconjugate vaccine, Physicochemical characterization, ST131

## Abstract

Extraintestinal pathogenic *Escherichia coli* (ExPEC) cause a wide range of clinical diseases such as bacteremia and urinary tract infections. The increase of multidrug resistant ExPEC strains is becoming a major concern for the treatment of these infections and *E. coli* has been identified as a critical priority pathogen by the WHO. Therefore, the development of vaccines has become increasingly important, with the surface lipopolysaccharide constituting a promising vaccine target. This study presents genetic and structural analysis of clinical urine isolates from Switzerland belonging to the serotype O25. Approximately 75% of these isolates were shown to correspond to the substructure O25B only recently described in an emerging clone of *E. coli* sequence type 131. To address the high occurrence of O25B in clinical isolates, an O25B glycoconjugate vaccine was prepared using an *E. coli* glycosylation system. The O antigen cluster was integrated into the genome of *E. coli* W3110, thereby generating an *E. coli* strain able to synthesize the O25B polysaccharide on a carrier lipid. The polysaccharide was enzymatically conjugated to specific asparagine side chains of the carrier protein exotoxin A (EPA) of *Pseudomonas aeruginosa* by the PglB oligosaccharyltransferase from *Campylobacter jejuni*. Detailed characterization of the O25B-EPA conjugate by use of physicochemical methods including NMR and GC-MS confirmed the O25B polysaccharide structure in the conjugate, opening up the possibility to develop a multivalent *E. coli* conjugate vaccine containing O25B-EPA.

**Supplementary Information:**

The online version contains supplementary material available at 10.1007/s10719-021-09985-9.

## Introduction

Extraintestinal pathogenic *Escherichia coli* (ExPEC) are the most common Gram-negative bacteria causing disease like urinary tract infections (UTI), bacteremia and meningitis [1]. It is the main cause of bacteremia in elderly and UTI in young healthy women [2]. The increase of antibiotic resistance has become a major concern in the treatment of infections caused by *E. coli*. The multidrug-resistant (MDR) clonal lineages might lead to mortality and morbidity as in the pre-antibiotic area. The *E. coli* O25B sequence type 131 (ST131) has been identified as one of the worldwide pandemic strains that causes predominantly community acquired infections [3]. It was first described in 2008 [4, 5] and accounts for more than 10% of all ExPEC infections and is the major cause of serious multidrug-resistant *E. coli* infections in the United States [6]. Recently, it was not only shown that the O25B differed in its O antigen gene cluster from the previously described O25A strain [7], but also in its structure [8].

The O antigens are the dominant features of the *E. coli* bacterial cell surface and constitute important determinants of virulence and pathogenicity [9]. The O antigen is part of the lipopolysaccharide (LPS) that is composed of four structural entities: the lipid A is embedded in the outer membrane and consists of branching acyl chains held together by two glycosidically linked glucosamine residues. The conserved monosaccharides (Kdo2-Hep2) of the inner core are linked to the non-reducing end of lipid A. A variable outer core decorates the inner core. The O antigen is linked to the outer core, forming the complete LPS structure [10]. The O antigen of *E. coli* is the most diverse structure, with over 180 individual serotypes, attributed to unique O antigen structures [9]. In humans, O antigens contribute to *E. coli* survival through the evasion of host defenses [11]. In patients with bacteremia, antibody response is mainly directed against O antigens, and therefore, the O antigen seems to be suitable for the development of an ExPEC vaccine [12].

Conjugate vaccines are the most efficient way to prevent infections caused by bacteria. They have been licensed to prevent infections caused by *Streptococcus pneumoniae*, *Neisseria meningitidis*, *Haemophilus influenzae* type b [13] and *Salmonella typhi* [14]. All these conjugates consist of capsular polysaccharides chemically conjugated to carrier proteins. A multivalent *E. coli* O antigen based conjugate vaccine has been shown to be safe and immunogenic in humans [15]. However, the conventional production of such O antigen derived multivalent conjugate vaccines is more complex than for capsular polysaccharide conjugate vaccines; the O antigen preparation requires laborious extraction of the LPS, chemical release of the O antigen and detoxification procedures. In addition, the chemical detoxification might impact on the structural integrity of the O antigen.

An innovative *E. coli* glycosylation technology simplifies the development of a multivalent LPS based *E. coli* conjugate vaccine [16, 17]. It is based on a general N-linked glycosylation system engineered in *E. coli* that allows the site directed enzymatic conjugation of O antigens to carrier proteins, generating bioconjugates. An important aspect in the development of a multivalent conjugate vaccine is the correct identification of O antigen structures in clinical isolates. However, identification of serotypes based on typing sera can lead to wrong serotype attribution, as these polyclonal sera often give unspecific response. We have investigated the serotype distribution of bacterial clinical isolates from women having UTI. More than 20% of the strains were agglutinated with the polyclonal typing sera raised against whole cell extracts of *E. coli* O25A. Further structural analysis identified that more than 75% of these isolates belong to the O25B subserotype. Sequence analysis of the O antigen cluster confirmed the O25B composition [18].

Following identification of the prevalence of *E. coli* O25B, we describe the production of a novel O25B conjugate vaccine achieved by genetically engineering an *E. coli* protein glycosylation system using a genuine O25B O antigen cluster. The structure of the O-acetylated pentasaccharide repeating unit (RU) was demonstrated using composition and linkage analysis, mass spectrometry and NMR spectroscopic studies.

## Materials and methods

### Bacterial strains, plasmids and reference materials

#### Bacterial strains and plasmids

*E. coli* strains were grown in LB at 37 °C. Kanamycin (Kan), 50 μg/mL; tetracycline (Tet), 20 μg/mL; Spectinomycin (Sp), 80 μg/mL; Chloramphenicol (Clm), 20 μg/mL; and Ampicillin (Amp), 100 μg/mL were added to the media for selection as needed. *E. coli* DH5α (Life Technologies, Carlsbad, CA, USA) was the host for cloning experiments. Plasmids pEXT21 and pBR322 (NEB, Beverly; MA; USA) were used as cloning vectors. *E. coli W3110* was obtained from the Coli Genetic Stock Center, Yale University, New Haven, CT, USA. Deletion of the *waaL* chromosomal gene in W3110 was performed according to Datsenko and Wanner as described [19]. Plasmids p150 [20] and p114 [21] have been described elsewhere. The different *E. coli* strains were from an epidemiology study from women with UTI (GlycoVaxyn AG, unpublished data). Strains, plasmids and primers used are listed in Table S1.

#### Construction of plasmids

For the construction of shuttle plasmids that deliver the O25B *rfb* cluster for integration, the *rfb* cluster of a clinical isolate with O25B positive LPS phenotype (strain UPEC138, [22]) was amplified using oligonucleotides 2025 and 2266, and cloned into pDOC-C [23]. *E. coli* strain DH5α was used for DNA cloning experiments and constructed plasmids were verified by DNA sequencing.

#### Construction of recombinant *E. coli* W3110 cells expressing the O25B antigen

For chromosomal replacement of the W3110 O16 *rfb* cluster, a method specifically developed for this purpose was applied [24]. For deletion of chromosomal copies of the *waaL* gene and the *gtr*ABS cluster, the method for homologous recombination followed by FLP mediated removal of the resistance cassette by Datsenko and Wanner was used [25]. Chromosomal genotypes were confirmed by colony PCR and whole genome or Sanger sequencing. Phenotypes were analyzed by LPS or lipid-linked oligosaccharide western blotting, silver staining, and 2-AB labelling as described later.

#### Production of O25A and O25B LPS and O-PS reference material

*E. coli* strain O25A (UPEC436, [22]) and *E. coli* strain O25B (UPEC177, [22]) were grown overnight in LB at 37 °C and LPS extracted by the method of Apicella [26] as described [19].

### Production and purification of O25A-EPA and O25B-EPA

Bioconjugates were produced either in wild-type *E. coli* strains sourced from an epidemiology study conducted in Switzerland (GlycoVaxyn AG, Switzerland, unpublished data) or in a recombinant strain expressing O25B O antigen. Modified clinical isolates (*E. coli* UPEC438 *rfb*O25A; Δ*waa*L; p112 (*pgl*B); p659 (EPA)) were used for O25A-EPA conjugate production, and recombinant *E. coli* W3110 (Δ*gtr*ABS; Δ*rfb*W3110::*rfb*O25B; Δ*waa*L; p970 (*pgl*B) and p1076 (EPA)) for O25B-EPA. The isolates were grown in a fed-batch fermentation process in a phosphate-buffered complex media as described previously [27]. Appropriate amounts of antibiotics and inducer were added to the feed. The fermentation media of the recombinant *E. coli* strain was furthermore supplied with trace elements to compensate for potential fluctuation in complex media composition. Biomass was harvested between 15 and 34 h post induction when product formation reached a maximum. The O25A-EPA bioconjugate produced in wild-type *E. coli* was purified from periplasmic extracts by osmotic shock, and column purification as described [28]. Final bulk was formulated in Tris-buffered saline (25 mM Tris pH 7.4, 137 mM NaCl, 2.7 mM KCl). The resulting bioconjugates were analytically characterized for content, purity and impurities to demonstrate suitability for preclinical and extended structural studies.

### SDS-PAGE, silver staining, and immunoblotting

Bacterial cell extracts were lysed in Lämmli buffer and separated by SDS-PAGE. For protein analysis, NuPAGE™ Novex™ 3–8% Tris-acetate protein gels (Thermo Fisher Scientific, Waltham, MA, USA) were used and either stained with Colloidal Blue (Thermo Fisher Scientific) or subjected to western blotting. For the analysis of glycolipids (LPS or LLO), NuPAGE™ Novex™ 12% Tris-acetate gels (Thermo Fisher Scientific) were used and extracts were additionally treated with Proteinase K (1 g/L) for 1 h at 37 °C after lysis in Lämmli buffer to suppress protein signals. For immunoblotting, samples were transferred using an iBlot® 2 Dry Blotting System (Thermo Fisher Scientific) followed by immunostaining. Polyclonal anti-*Pseudomonas* Exotoxin A antibody produced in rabbit (Sigma-Aldrich, Switzerland), a polyclonal rabbit anti-*E.coli* O25 antiserum (Denka Seiken, Tokyo, Japan), or custom made antisera were used for immunoblotting as described [17]. For signal visualization, secondary goat anti-rabbit IgG-horse radish peroxidase conjugate (Sigma-Aldrich) in combination with NBT/BCIP detection kit (Roche, Switzerland). Agglutination assays were performed as described previously (DebRoy *et al*. Dec).

### Physicochemical analysis

#### Analysis of undecaprenyl-pyrophosphate (UPP)-linked polysaccharides

UPP-linked O-polysaccharides were extracted, hydrolyzed, purified, labeled with 2-aminobenzamide (2-AB), separated by normal phase HPLC and analyzed by MALDI MS/MS as described by Wetter *et al*. [29], except that tetrabutylammonium phosphate was omitted during purification.

#### Monosaccharide compositional analysis by RP-HPLC

10 μg of polysaccharide of O25A-EPA and O25B-EPA were hydrolyzed by trifluoroacetic acid (TFA) and the released monosaccharides were labeled by reductive amination with 1-phenyl-3-methyl-2-pyrazoline-5-one (PMP). The derivatized monosaccharides were solvent extracted, analyzed by C_18_ RP-HPLC and detected by UV at 250 nm as described [19]. Monosaccharides treated the same way were used as standards to confirm monosaccharide identity.

#### Monosaccharide compositional analysis and absolute configuration determination by GC

The monosaccharide composition of O25B O-PS (500 μg) and O25B-EPA (400 μg saccharide) was determined by GC and GC-MS after derivatization of the samples to tri-methyl-silyl ethers methyl-glycosides (methyl-glycosides TMS). The samples were prepared by methanolysis (3 M HCl for 16 h at 85 °C) and derivatized following the method described by Kim *et al*. [30]. After re-N-acetylation of the amino sugars with acetic anhydride, the resulting methyl glycosides were TMS-derivatized using the silylating reagent (HMDS+TMCS+Pyridine, 3:1:9 (Sylon HTP) Kit from Supelco). The samples were dried under a stream of N_2_, hexane was added, followed by centrifugation to remove insoluble material. The clear supernatants were dried under N_2_, the samples dissolved in hexane and analyzed either by GC on a Perkin–Elmer Autosystem XL gas chromatograph equipped with a flame ionization detector and a capillary HP-1 column, using He as carrier gas or by GC-MS on an Agilent Technologies 7890A gas chromatograph coupled to an Agilent Technologies 5975C VL MSD using the same column. The temperature program was: 150 °C for 1 min, 150–280 °C at 3 °C/min, and then held at 280 °C for 2 min. Standard retention times and response factors for the TMS methyl glycosides were determined using monosaccharides standards and the internal standard inositol. A sample of O-PS from *Yersinia enterocolitica* O:50 strain 3229 [31] was used to obtain a standard for L-FucNAc; identification of the peaks attributable to L-FucNAc was confirmed by GC-MS.

Determination of the absolute configuration of the monosaccharide residues in O25B-EPA was performed according to Gerwig *et al*. [32, 33]. 500 μg of each monosaccharide standard (all with the D configuration, except L-Rha) was subjected to butanolysis in 1 M HCl in S-(+)-2-butanol or R-(−)-2-butanol for 16 h at 80 °C. Re-N-acetylation, TMS-derivatization of the free hydroxyl groups and purification were performed as described above for the preparation of the TMS methyl-glycosides. The monosaccharide standards were used to prepare S-(+)-2-butyl-glycoside TMS as well as R-(−)-2-butyl-glycoside TMS standards for each monosaccharide. The O25B-EPA sample (400 μg) was subjected to methanolysis (3 M HCl, 16 h at 85 °C) followed by butanolysis using S-(+)-2-butanol and derivatization as described for the monosaccharide standards. Attribution to the D- or L- absolute configuration was achieved by comparing the elution time of the samples with those of the monosaccharide standards. The GC temperature program was: 50 °C for 1 min, 50–130 °C at 45 °C/min, at 130 °C for 1 min, 130–200 °C at 1 °C/min, and finally held at 200 °C for 10 min. GC-MS was used to confirm the data obtained with GC and to identify all peaks present in the chromatograms.

#### Quantification of O-acetyl content

The O-acetyl content of O25B-EPA samples was determined by high performance anion exchange chromatography with conductivity detection (HPAEC-CD) following release of the O-acetyl groups by use of mild alkaline hydrolysis. Conjugate samples (saccharide concentration ~ 100 μg/mL) were desalted on a Zeba™ Spin Desalting column featuring a 7 kDa cut off (Thermo Fisher Scientific) and hydrolyzed in 10 mM NaOH (4 h at 37 °C) in the presence of propionate serving as internal reference standard. The resulting samples were loaded to centrifugal filter devices with a MW cut off of 3 kDa (Merck, Darmstadt, Germany) and spun once at 16,000×g for 15 min. Permeates containing released acetate were analyzed by HPAEC-CD on a Summit HPLC system (Thermo Fisher Scientific) equipped with an ED50 conductivity detector and an auto-suppression recycling suppressor ASRS 300. Chromatography was performed on an IonPac AS11-HC column (Thermo Fisher Scientific) by isocratic elution with 1 mM NaOH over 15 min at a flow rate of 1.5 mL/min. Chromeleon V.6.80 SR9 software was used for data analysis. The amount of acetate was determined via a standard curve of 1–10 μg/mL acetate and the extent of O-acetylation in conjugate samples calculated based on the mass proportion of acetate to the polysaccharide RU.

#### Monosaccharide linkage analysis by GC-mass spectrometry

Prior to linkage analysis the conjugates were digested to glycopeptides by the classical trypsin treatment followed by purification on an ENVI-Carb column (Supelco). Permethylation of the O25B glycopeptide sample (500 μg), was achieved following the method described by Harris *et al*. [34]. After organic extraction, the permethylated polysaccharides were hydrolyzed with 4 M TFA (1 h at 125 °C) followed by derivatization to alditol acetates as described by Albersheim *et al*. [35]. The resulting partially methylated alditol acetates (PMAA) derivatives were purified by CHCl_3_ extraction and analyzed by GC and GC-MS using an HP-1 capillary column and the temperature program: 120 °C for 1 min, 120–245 °C at 2 °C/min, and held at 245 °C for 20 min. Identification of the sugar type followed from retention times, while the ring size and the glycosidic linkage positions were determined from the corresponding mass spectra. Quantification of each sugar derivative was achieved by correcting the corresponding area obtained in the gas chromatogram by the effective carbon response factor according to Sweet *et al*. [36].

#### NMR spectroscopy

NMR samples were either prepared as intact glycoconjugates by re-buffering into water or as glycopeptides prepared by Pronase E (Sigma-Aldrich) digestion and subsequent purification on Supelclean™ ENVI-carb™ SPE tubes (Sigma-Aldrich) and PD10 (GE Healthcare Life Sciences) desalting columns. Glycoconjugate or glycopeptide samples (~1 mg polysaccharide) were lyophilized and exchanged twice with 99.9% deuterium oxide (Sigma Aldrich), then dissolved in 600 μL of D_2_O and introduced into a 5 mm NMR tube (Wilmad®, Sigma Aldrich) for data acquisition. The intact LPS sample (37 mg) was dissolved in D_2_O and analyzed without deuterium exchange. Spectra were also recorded on the O25B glycopeptide sample after the addition of sodium deuteroxide to a final concentration of 200 mM (in order to achieve de-O-acetylation in the NMR tube). 1D ^1^H and 2D, COSY, TOCSY, NOESY, HSQC, HMBC and hybrid HSQC-TOCSY NMR spectra were obtained using a Bruker Avance III 400 NMR with an Ultra Shield 400 Plus magnet and processed using standard Bruker software (Topspin 3.2). A sample of O25B-EPA (4.5 mg) was also analyzed using a Bruker Avance III 600 MHz NMR spectrometer equipped with a BBO Prodigy cryoprobe. The probe temperature was set at 303, 313 or 323 K. 2D TOCSY experiments were performed using mixing times of 120, 180 or 200 ms and the 1D variants using mixing times of 200 or 250 ms. The HSQC experiment was optimized for J = 145 Hz (for directly attached ^1^H-^13^C correlations), and the HMBC experiment optimized for a coupling constant of 8 Hz (for long-range ^1^H-^13^C correlations). HSQC-TOCSY and HSQC-NOESY NMR spectra were recorded using mixing times of 120 and 250 ms respectively. Spectra were referenced to residual acetate (^1^H signal at 1.903 ppm and ^13^C signal at 23.97 ppm [37]) or acetone added as an internal standard (^1^H signal at 2.225 ppm and ^13^C signal at 31.45 ppm).

## Results

### Analysis and preliminary characterization of O25A and O25B O antigen polysaccharide structures found in clinical O25 isolates

**Fig. 1:**
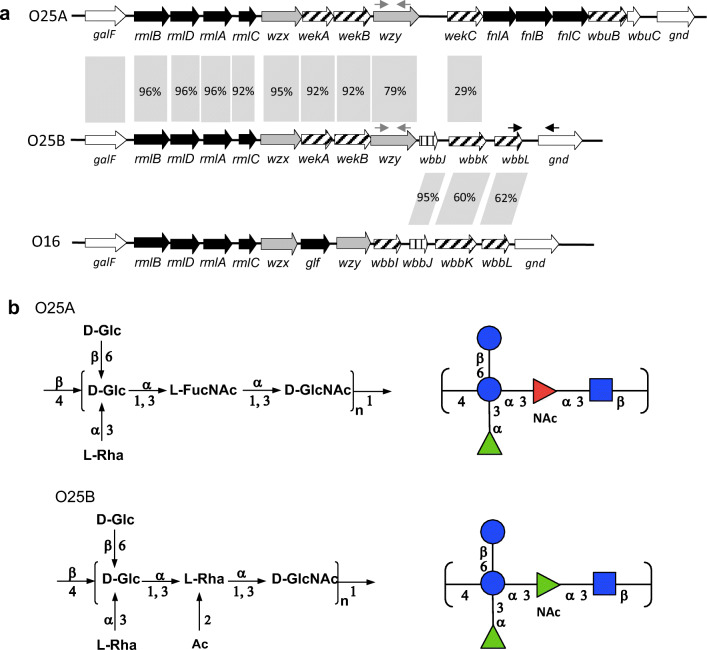
Gene clusters and corresponding structures of *E. coli* O25A and O25B subtypes. **a** Gene clusters encoding the synthesis of the O25A, O25B and O16 O antigens. Sequence identity values between genes of the three clusters are indicated in grey boxes where DNA sequences are related. Filling of the block arrows indicate gene functions (solid black: nucleotide activated monosaccharide biosynthesis; grey: lipid linked repeat unit flipping, *wzx*, and repeat unit polymerization, *wzy*; diagonally striped: glycosyltransferase; vertically striped: O acetyltransferase; no fill: unknown or outside the *rfb* cluster). Grey and black thin arrows indicate oligonucleotides used for O25A and O25B subtype specific typing PCR. **b** Chemical structures of the O25A and B O antigen RUs are shown (explicit and in CFG nomenclature). Brackets indicate the RU

**Fig. 2:**
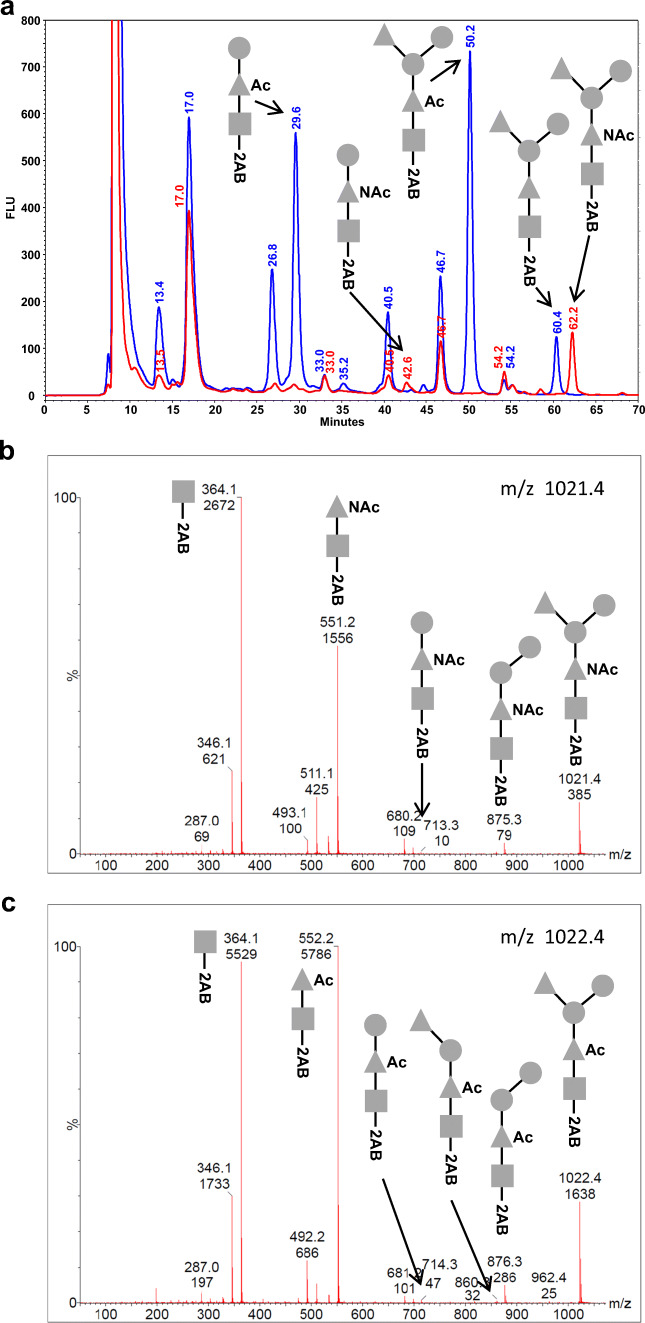
Analysis of UPP linked O25A and O25B O antigen by normal phase HPLC and mass spectrometry. **a** Oligosaccharides linked to UPP were extracted from *E. coli* biomass, released from UPP by mild-acid hydrolysis, reducing end labeled by 2-AB and analyzed by normal phase HPLC using fluorescence detection. An overlay of the elution spectrum up to 70 min is shown for extracts from clinical isolates UPEC436 (O25A, red) and UPEC138 (O25B, blue). A selection of strain specific peak fractions were collected and analyzed by MALDI MS/MS. Circle symbol corresponds to hexose (Hex), triangle to deoxy-hexose (dHex) and square to N-acetyl hexosamine (HexNAc). **b** MS/MS spectrum of m/z 1021.4 from fractions containing the peak eluting at 62.2 min (red trace from panel A). Fragmentation ions compatible with the O25A RU (dHex-Hex-Hex-dHexNAc-HexNAc-2-AB) were identified and labelled with the corresponding structure. **c** MS/MS spectrum of m/z 1022.4 from the fractions containing the peak eluting at 50.2 min (blue trace from panel A). Fragmentation ions compatible with the proposed O25B RU structure (dHex-Hex-Hex-dHexOAc-HexNAc-2-AB) are indicated (CFG nomenclature)

First, to construct an *E. coli* vaccine production strain, we needed to identify a gene cluster capable of expressing the target O25B O antigen. Multiple O25 agglutination positive clinical isolates were available to us from an epidemiology study (GlycoVaxyn AG, unpublished). Two O25A and O25B expressing strains were identified by PCR typing followed by full sequencing of the *rfb* cluster encoding the O antigen machinery in *E. coli*. As expected, the two sequences were matching for the genes encoding identical functionalities, while diverging for their structural differences as described (Fig. 1a). To confirm the expected differences (Fig. 1b) in the O antigens expressed by those strains, we analyzed their structures in detail. To do so, we took advantage of a method allowing O antigen structure analysis on its natural biosynthetic precursor: the membrane lipid undecaprenyl pyrophosphate (UPP). O antigen on gram negative bacteria is built up on both sides of the cytoplasmic membrane in a highly regulated process while attached to UPP. After synthesis, the O antigen is transferred to lipid A constituting lipopolysaccharide. We used a high resolution analysis method that enabled us to analyze the UPP-bound O antigen by HPLC and MS/MS [29]. Thereby, UPP-linked polysaccharides are extracted from cells, released from UPP by acid treatment and fluorescently labelled for detection after separation by normal phase HPLC. Figure 2a shows an overlay of the HPLC traces obtained from strains UPEC436 (O25A, red) and UPEC138 (O25B, blue). Fractions containing fluorescent material specific to one of the two extracts at 62.2 (O25A) and 50.2 (O25B) min, respectively, were analyzed by MALDI MS/MS. The fraction eluting at 62.2 min revealed a parent ion at m/z 1021.4 and a fragmentation series pattern which is in agreement with the RU of the published O25A repeat unit structure containing a 2-AB modification at the reducing end (Fig. 2b). The fraction eluting at 50.2 min from the O25B extracts showed a parent ion at m/z 1022.4 (Fig. 2c), and a different fragmentation pattern indicating a mass difference of one Dalton allocated to the second monosaccharide of the RUs. Taken together, the different HPLC elution profiles, the different masses of the RUs and their fragmentation patterns support the difference in the O antigen structures of *E. coli* O25 [8, 38], as predicted by the different enzymes encoded by the two gene clusters and their respective RU structures (Fig. 1a and b).

### Production of O25B-EPA bioconjugates and monosaccharide composition analysis

Due to the high prevalence of the O25B subserotype in clinical isolates causing disease, we aimed at producing an O25B conjugate vaccine component to be included in a multivalent *E. coli* vaccine using the *E. coli* bioconjugation technology [16, 17]. Therefore, we constructed an *E. coli* W3110 cell line capable of synthesizing a bioconjugate consisting of the O25B O antigen covalently linked to the carrier protein EPA, a recombinant detoxified *Pseudomonas aeruginosa* exotoxin A, using enzymatic conjugation by PglB [17].

**Fig. 3:**
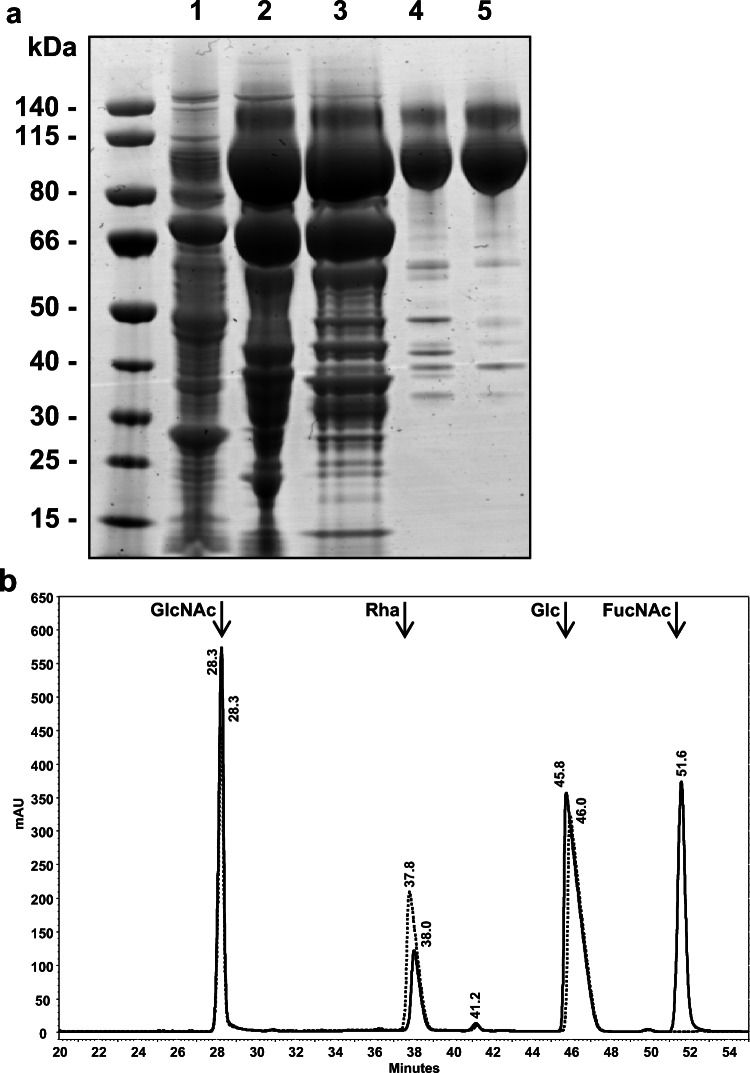
Purification and analysis of O25B-EPA. **a** O25B-EPA conjugate was purified from *E. coli* cells expressing the O25B polysaccharide, EPA and PglB. The purified conjugate was separated by SDS-PAGE and visualized by Colloidal blue staining. Lane 1 represents the homogenized cell substrate after clarification, lane 2 the pooled eluate after Pall Q purification, lane 3 the pooled eluate after Butyl-Sepharose purification, lane 4 the pooled eluates after Source Q purification and lane 5 the pooled eluates after Superdex-200 purification corresponding to the final bulk. The molecular weight of the Page ruler protein standards (Thermo Fisher Scientific) is given on the left. **b** Both O25-EPA conjugates and monosaccharide standards were hydrolyzed, labeled with PMP, purified by organic extraction and analyzed by C_18_ RP-HPLC. Arrows indicate the elution time of monosaccharide standards detected at 250 nm. The solid line represents the hydrolyzed monosaccharides from O25A-EPA, the dotted line the ones from O25B-EPA

In a first step, the gene cluster of UPEC138 capable of synthesizing O25B as shown above was inserted into the production strains W3110 by replacing its endogenous O antigen cluster by homologous recombination. The W3110 strain contains a prophage encoded *gtr*ABS operon that adds a branching glucose residue to the O16 O antigen in the periplasm. To avoid the risk of an unexpected modifications of the O25B polysaccharide during recombinant expression, the *gtr*ABS cluster was deleted as well as the gene encoding the ligase WaaL, resulting in an *E. coli* W3110 accumulating the O25B antigen on the carrier lipid UPP. To transfer the polysaccharide antigen to EPA, plasmids expressing EPA and PglB under inducible promotors were transformed into the chromosomally tailored O25B expression strain. The resulting production strain was grown at 35 °C and expression of PglB and EPA was induced. Proteins were extracted and the bioconjugate was purified as described in experimental procedures section. Representative intermediate pools from the purification procedure were analyzed by SDS-PAGE (Fig. 3a). Bioconjugate vaccine O25B-EPA containing the O25B polysaccharide antigen is indicated by the ladder like distribution of the protein signal in lane 5 at molecular weights corresponding to 80–140 kDa, and confirmed by western blotting (not shown).

To verify the polysaccharide structure of the bioconjugate produced recombinantly and to compare its structure to the lipid-linked polysaccharides from the clinical isolates (Fig. 2), monosaccharide compositional analysis was performed on the purified conjugate as previously described [19]. Figure 3b shows elution time of the monosaccharide standards as well as hydrolyzed O25B-EPA (dotted line). Peaks were observed for the monosaccharide standards Glc, GlcNAc, and Rha and for the EPA-O25B in agreement with the result of the lipid-linked polysaccharides analysis from clinical isolates. In contrast, the monosaccharide analysis of O25A-EPA produced using a clinical O25A isolate deleted for *waaL* and recombinantly expressing of PglB and EPA showed peaks (solid line) for the four monosaccharides Glc, GlcNAc, FucNAc and Rha, in agreement with the analysis of lipid-linked polysaccharides and the published RU structure of *E. coli* O25A LPS [38]. These results further confirm our antigenic and genetic differences observed between O25A and O25B. The harsh hydrolysis conditions employed released the amino sugars that were key to differentiating between O25A and O25B, but also resulted in low recovery for Rha due to degradation of the free 6-deoxy hexoses in acid. Therefore composition analysis was also performed using methanolysis which releases the monosaccharides as the relatively stable methyl glycosides. The disadvantage of methanolysis is the presence of multiple peaks due to α- and β-anomers of the pyranose and furanose ring forms for each monosaccharide; this was addressed by preparing methanolysis standards of each monosaccharide alone and in the expected mixture. The presence of Rha, Glc and GlcNAc in O25B-EPA was confirmed by GC analysis of the derived TMS methyl glycosides and use of response factors gave the monosaccharide molar ratio of 2.20: 2.00: 0.84, respectively, consistent with the RU of *E. coli* O25B. GC analysis of the LPS sample yielded a similar chromatogram proving that the O25B RU expressed by the *E. coli* O25B wild-type isolate was successfully expressed by the recombinantly-engineered host cells. With the sugar composition established, the absolute configuration of the constituent monosaccharides was investigated. GC analysis of the S-(+)-2-butyl glycosides TMS derivatives from O25B-EPA indicated the presence of L-Rha, D-Glc and D-GlcNAc, in agreement with the putative *E.coli* O25B antigen structure. This proves that the second Rha present in the O25B serotype, instead of FucNAc present in O25A, must also have the L-configuration. The identity of the sugar-derived peaks was confirmed by GC-MS analysis. Having established the ratio and identity of the constituent monosaccharides and their absolute configurations, the presence of non-sugar components was investigated.

### Determination of O-acetyl content of O25B-EPA

The presence of O-acetylation in the O25B O antigen RU predicted by the homology to O16 was first shown by analysis of the 2-AB labelled oligosaccharide (Fig. 2c) and its presence and location at C-2 of the 3-linked Rha was subsequently elucidated by NMR analysis. A composition quantification assay was developed to show that O-acetylation was expressed by the recombinantly-engineered host cells and that it was not removed during subsequent O25B-EPA conjugate isolation and purification. The O-acetyl content of O25B-EPA and O25A-EPA (as a control) was determined by HPAEC-CD after desalting (to remove free acetate) and release of attached O-acetyl groups by mild alkaline treatment. The degree of O-acetylation, calculated per RU, was 98% for O25B-EPA and below the detection limit of the assay for O25A-EPA (Table S2), which is in agreement with the degree of O-acetylation determined by NMR analysis (see below).

### Linkage analysis establishes the monosaccharide pyranosyl ring forms and linkage positions of the O25B RU

The linkages of the constituent monosaccharides was investigated by use of methylation analysis. GLC analysis of the partially-methylated alditol acetate (PMAA) derivatives (Fig. S1) showed that O25B contains terminal Rha*p* (t-Rha*p*), 3-linked Rha*p* (3-Rha*p*), terminal glucose (t-Glc*p*), 3,4,6-linked Glc*p* (3,4,6-Glc*p*) and 3-linked Glc*p*NAc (3-GlcNAc*p*) in the molar ratio 0.48: 1.00: 0.95: 0.72: 1.35. The assignments were based on relative retention times and GLC-MS-e.i. spectra. These data are consistent with the published O25B pentasaccharide RU proposed from structural studies and the NMR characterization of the RU attached to the core oligosaccharide [8].The small peak due to 3,6-Glc (0.09), was attributed to the non-reducing end of the polysaccharide present in the glycopeptide preparation, this was confirmed by NMR studies. The peak attributed to 4,6-Glc (0.47) is probably due to loss of the t-Rha residue during the permethylation reaction, as shown also by the amount of t-Rha detected (0.48), less than the expected value of 1.0. Estimation of the average chain length of the RU can be made by addition of the in-chain Glc (4,6-Glc and 3,4,6-Glc = 1.19) divided by the molar ratio of non-reducing terminal Glc (0.09 for 3,6-Glc) which gives 13.2, which is in good agreement with the average chain length of 11–17 RU estimated from analysis of the 2-AB-linked glycans released by hydrazinolysis (data not shown). Although 4-Rha was detected in small amounts, it is not part of the reported RU, and its origin cannot be explained, while the 2,3-Rha might arise from undermethylation. Ester linked O-acetyl groups are lost during the treatment with strong base required for linkage analysis and therefore its location on C-2 of the 3-linked Rha was addressed by NMR analysis.

### NMR characterization of O25B-EPA and the corresponding glycopeptide

1D and 2D NMR analysis was performed in order to elucidate the O25B RU structure in the O25B-EPA bioconjugate vaccine. Studies were performed on the O25B-EPA glycoconjugate and the corresponding glycopeptide as well as intact LPS. An overlay of the ^1^H NMR spectrum of O25B-EPA compared to O25A-EPA revealed key differences between the RU structures: the absence of signals ascribed to 3-linked α-FucNAc (H-1 at 4.97 ppm, an N-acetyl signal at 1.96 ppm and an upfield doublet from H-6 at 1.16 ppm) and a new set of signals which were subsequently assigned to 3-linked α-Rha2Ac (Fig. S2). The presence of O-acetylation of O25B was demonstrated by an O-acetyl signal at 2.13 ppm and the presence of the deshielded ring proton in the anomeric region of the spectrum (H-2 at 5.21 ppm, Fig. S2).

**Fig. 4:**
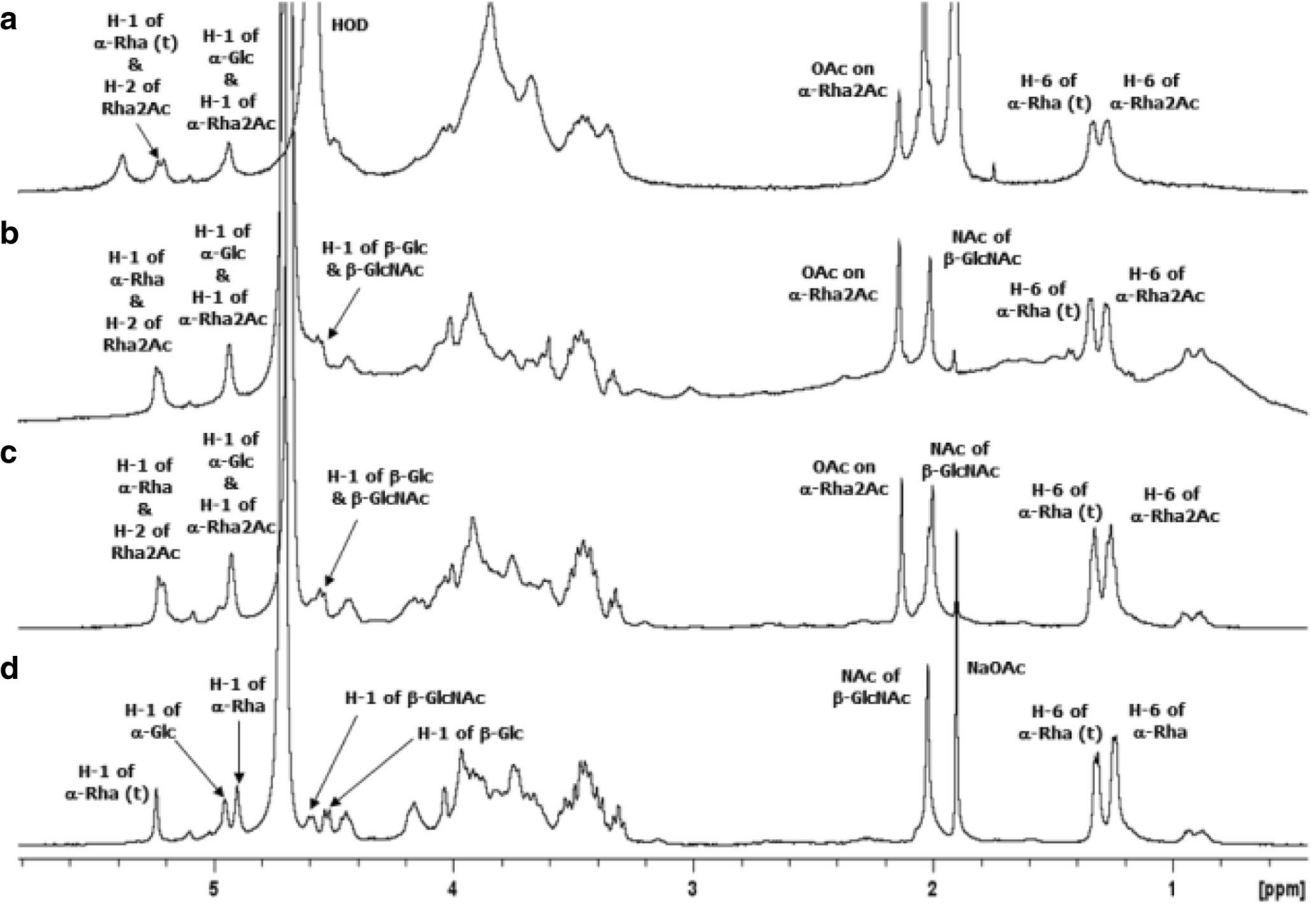
Overlay of ^1^H NMR spectra of: **a** intact LPS isolated from *E. coli* O25B, **b** O25B-EPA conjugate, **c** the glycopeptide O25B-GP, and **d** the de-O-acetylated O25B-GP (NaOD) sample. The spectra were recorded at 400 MHz at 303 K for all samples except for the LPS (313 K)

NMR analysis of the intact LPS isolated from wild type *E. coli* O25B revealed the same diagnostic signals present in O25B-EPA attributed to the pentasaccharide RU containing 3-linked α-Rha2Ac (Fig. 4a, b). Additional signals present in the NMR spectrum of LPS were due to lipid (methyl and methylene signals) and a co-purified surface glycan (anomeric signal near 5.4 ppm).

**Fig. 5:**
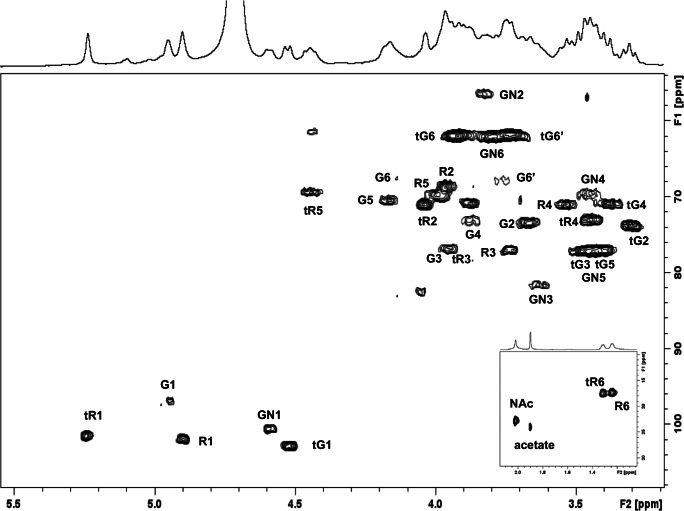
Expansion of the HSQC spectrum of O25B-GP (NaOD) recorded at 400 MHz, the crosspeaks from the methyl region of the spectrum are shown in the inset. Key pentasaccharide RU proton/carbon crosspeaks have been labeled according to the carbon atom of the corresponding residue (R = Rha, G = Glc and GN = GlcNAc, with the terminal sugars indicated by r). Small peaks are due to residual buffer and amino acids

Detailed NMR studies were performed on the O25B glycopeptide (O25B-GP) obtained by pronase treatment of the O25B-EPA conjugate and purified by reversed-phase and size-exclusion chromatography. The proton NMR spectrum of O25B-GP (Fig. 4c) contained the same carbohydrate signals as the parent conjugate (Fig. 4b) demonstrating that the pronase treatment had no impact on the structural integrity of the O25B antigen. Due to the spectral complexity introduced by O-acetylation, the structure of the O25B RU sugar backbone was first elucidated by analysis of the corresponding de-O-acetylated sample (achieved by the addition of NaOD to the NMR tube). The ^1^H NMR spectrum of the de-O-acetylated O25B-GP exhibited five major anomeric signals at 5.24, 4.95, 4.90, 4.59 and 4.53 ppm assigned to three α-linked and two β-linked residues respectively, an N-acetyl signal at 2.02 ppm and two upfield doublets at 1.31 and 1.24 ppm due to H-6 of the two α-Rha residues (Fig. 4d). The anomeric and H-6 signals served as the starting points for the 2D ^1^H-^1^H (COSY and TOCSY), 2D ^1^H-^13^C direct (HSQC) and long range (HMBC) correlation experiments which permitted full elucidation of the terminal α-Rha (t) and β-Glc, 3,4,6-linked α-Glc and 3-linked β-GalNAc spin systems (as found in O25A) and established the new residue in O25B as 3-linked α-Rha. The COSY/TOCSY overlay elucidating the α-Rha spin systems is shown in Fig. S3A and the HSQC in Fig. 5; the NMR data are collected in Table 1. The deshielded carbons C-3, C-4 and C-6 of α-Glc, C-3 of β-GlcNAc and C-3 of α-Rha confirmed the linkage positions, whereas the sequence of sugar residues indicated by glycosylation shifts followed from the HMBC interresidue correlations and negative peaks in the TOCSY experiments, attributed to dipolar coupling. Thus NMR analysis confirmed the structure of the pentasaccharide RU of the O-PS *E. coli* O25B as →4)-[β-D-Glc*p*-(1 → 6)]-[α-L-Rha*p*-(1 → 3)]-α-D-Glc*p*-(1 → 3)-α-L-Rha*p*-(1 → 3)-β-D-Glc*p*NAc-(1→. The chemical shift assignments are in good agreement with the CASPER predictions for the O25B RU [39].

**Table 1:** NMR data of *E. coli* O25B (de-OAc) glycopeptide RU and Rha(2Ac) of native O25B (lower panel)

Residue	H-1	H-2	H-3	H-4	H-5	H-6	N- or O-acetyl		HMBC from H-1
	C-1	C-2	C-3	C-4	C-5	C-6	methyl	CO	
α-L-Rha*p-*(1→ (tR)	5.24	4.04	3.88	3.45	4.44	1.31			76.8 (C-3 of G)
	101.4	70.9	70.8	72.9	69.4	17.4			
3,4,6)-α-D-Glc*p*-(1→ (G)	4.95	3.67	3.95	3.87	4.17	4.15, 3.76			
	96.8	73.3	76.8	73.2	70.4	67.5			
3)-α-L-Rha*p-*(1→ (R)	4.90	3.96	3.74	3.54	4.00	1.24			81.5 (C-3 of GN)
	101.8	68.6	76.9	70.9	69.6	17.2			
β-D-Glc*p*-(1→ (tG)	4.53	3.30	3.48	3.38	3.45	3.94, 3.74			67.5 (C-3 of G)
	102.7	73.7	77.1	70.8	77.1	61.7			
3)-β-D-Glc*p*NAc-(1→ (GN)	4.59	3.83	3.63	3.45	3.44	~3.79	2.02		
	100.5	56.3	81.5	69.5	77.0	61.9	22.8	175.1	
3)-α-L-Rha*p*2Ac-(1→ (R2Ac)	4.93	5.21	3.93	3.60	4.07	1.26	2.13		
	99.4	69.0	73.3	70.9	69.4	17.1	21.0	173.6	

The spectra were recorded at 303 K (400 MHz) and referenced to residual sodium acetate (^1^H at 1.903 ppm and ^13^C at 23.97 ppm). Deshielded ring carbons (linkage positions) are underlined

NMR experiments performed on the O-acetylated glycopeptide O25B-GP confirmed the presence of 3-linked α-Rha2Ac with H-2 at 5.21 ppm (Fig. S3B); the NMR assignments for this residue are provided in Table 1, lower panel. These assignments were confirmed by detailed NMR analysis of O25B-EPA conducted at 600 MHz. The HSQC experiment revealed all the proton/carbon pairs for the O-acetylated pentasaccharide RU with diagnostic signals assigned to the five anomeric signals, H-2/C-2 of α-Rha2Ac (at 5.21/69.0 ppm), the O-acetyl and N-acetyl signals and the two H-6/C-6 signals for terminal α-Rha and 3-linked α-Rha2Ac. The assignments are in agreement with those published for the RU-core oligosaccharide [8] with small differences as the α-Glc is 3,6-linked not 3,4,6-linked as in the O25B RU. The glycosylation shifts due to O-acetylation accord with those reported for the 3-linked α-Rha2Ac present in the -α-D-Glc*p*-(1 → 3)-α-L-Rha*p*-(1 → 3)-β-D-Glc*p*NAc-(1 → sequence from *E. coli* K12 [40]. The small peak at 5.09 ppm is due to α-Rha linked to the terminal 3,6-linked α-Glc as elucidated for the published for the RU-core oligosaccharide [8]. In conclusion, NMR studies show that the structural integrity of the O-acetylated O-PS produced by wild type *E. coli* O25B is maintained throughout the biosynthesis and purification of the O25B-EPA glycoconjugate vaccine.

## Discussion

Extraintestinal pathogenic *E. coli* strains cause a wide range of disease ranging from UTI, bacteremia and meningitis. The increasing use of antibiotics has led to the emergence of multi-resistant strains and *E. coli* has been identified as a critical priority pathogen by the WHO. The paucity of new antimicrobials in the clinical pipeline resulted in the development of vaccines preventing these bacterial infections which have become a major medical need. Conjugate vaccines, containing capsular surface polysaccharides, chemically linked to protein carriers, have been the most successful vaccines to prevent various bacterial infections. However, these chemical conjugation methods present challenges to the development of LPS-based vaccines due to manufacturing complexity and the multivalency required. Such conjugation is hampered by the complexity of the involved chemistry, which is a combination of the diversity of the many different carbohydrate antigens, the sensitivity of immunologically relevant structures such as O-acetylation to chemical treatments, and the consequent development efforts required. A novel enzymatic bioconjugation technology in which the O-polysaccharide is assembled on its carrier lipid and enzymatically transferred to specific residues of the protein carrier via an N-glycosidic linkage has allowed the facile development of LPS-based conjugate vaccines [17, 19]. This technology has been applied to *Shigella* [41, 42] and has opened up the possibility to develop a multivalent *E. coli* vaccine that prevents infections caused by prevalent serotypes [43–45].

In this study we first established O25B and O25A structure relationships. We performed genetic analysis of clinical urine isolates from Switzerland typed as belonging to the serotype O25. Structural confirmation of the RU differences by HPLC profiling and mass spectrometry identified a critical mass unit difference of 1 Da, in agreement to the proposed RU structures for O25A and O25B represented by the respective O25A and O25B isolates UPEC436 and UPEC138.

The next stage involved preparation of the corresponding bioconjugates using the two O antigen clusters and *E. coli* W3110 to generate O25A-EPA and O25B-EPA respectively. The bioconjugates were amenable to structural characterization with O25A-EPA serving as a comparator in the analyses performed. Composition analysis following acid hydrolysis and HPLC or methanolysis and GLC both showed the presence of Rha, Glc and GlcNAc in O25B-EPA, and the absence of FucNAc (present in O25A-EPA). With the sugar composition established, the absolute configuration of the constituent monosaccharides was determined by GC analysis of the butyl glycoside derivatives to give L-Rha, D-Glc and D-GlcNAc, in agreement with the putative *E.coli* O25B antigen structure. The presence of an O-acetyl group per RU of O25B-EPA (absent for O25A-EPA) was determined by high performance anion exchange chromatography with conductivity detection (HPAEC-CD) following release of the O-acetyl groups by use of mild alkaline hydrolysis.

Finally more detailed chemical and spectroscopic characterization studies were performed on O25B-EPA and the corresponding glycopeptide. Linkage analysis involving methylation analysis and GLC analysis of the partially-methylated alditol acetate (PMAA) derivatives established the monosaccharide ring forms and linkage positions for the O25B RU: terminal Rha*p*, 3-linked Rha*p*, terminal Glc*p*, 3,4,6-linked Glc*p* and 3-linked Glc*p*NAc. 1D NMR analysis of O25B-EPA compared to O25A-EPA revealed key differences between the RU structures: the absence of signals ascribed to 3-linked α-FucNAc and new signals assigned to 3-linked α-Rha2Ac including an O-acetyl signal and a deshielded ring proton due to H-2 of α-Rha2Ac. These diagnostic signals were present in both the LPS isolated from wild type *E. coli* O25B and the corresponding glycopeptide prepared from O25B-EPA. Full NMR characterization was performed on the de-O-acetylated glycopeptide. A combination of ^1^H and ^13^C 2D NMR experiments identified the five sugar spin systems of the pentasaccharide RU and the linkage carbons, while the sequence was confirmed by HMBC interresidue correlations and negative peaks in the TOCSY experiments from dipolar coupling. Thus NMR analysis confirmed the structure of the O-acetylated pentasaccharide RU of the O-PS *E. coli* O25B as →4)-[β-D-Glc*p*-(1 → 6)]-[α-L-Rha*p*-(1 → 3)]-α-D-Glc*p*-(1 → 3)-α-L-Rha*p*2Ac-(1 → 3)-β-D-Glc*p*NAc-(1→. In conclusion the use of an array of physicochemical methods confirms the O25B polysaccharide structure in the recombinantly expressed bioconjugate O25B-EPA and opens up the possibility of developing a multivalent *E. coli* conjugate vaccine containing O25B-EPA.

This study has shown the way forward for the development and production of a multivalent *E. coli* vaccine. Compared to the multistep preparation of conjugate vaccines by chemical conjugation. Bioconjugation omits all chemical steps (and associated testing) by delegating the job to *E. coli* and a recombinant enzyme machinery. The carbohydrate antigen is biosynthetically produced by its genuine gene cluster, which can be – as we have shown here - isolated from the pathogen itself, or nowadays be synthesized if the sequence is known. Conjugation of protein carriers at defined sites is handled by a glycosylation enzyme, further avoiding random attachment or chemical modification of antigenic substituents that may result in the loss of labile groups such as O-acetylation. Further, we have demonstrated that the bioconjugate is amenable to direct high resolution characterization using physicochemical methods thus ensuring and controlling antigenic structure present on the vaccine even after conjugation, greatly reducing risk and the burden of manufacturing control.

Multiple human clinical trials using *E. coli* produced bioconjugate vaccines have been and are being conducted, showing that the vaccines were always safe and immunogenic [44, 45]. A multivalent vaccine candidate containing the here described O25B-EPA component was tested in human volunteers, shown to be safe and immunogenic, with a trend of efficacy [43]. Antibodies generated by this vaccine were also functional in opsonophagocytosis testings, showing that bioconjugates are able to raise antibodies possibly linked to efficacy [46]. Further development of a higher valency product is underway (NCT04306302 and NCT03819049 on www.clinicaltrials.gov).

Remarkably, it was also shown that the bioconjugation vaccine approach can protect from disease: a human challenge trial showed efficacy against experimental challenge with orally applied *Shigella flexneri* bacteria, using a bioconjugate vaccine containing the O antigen of the challenge [41]. Taken together, this data confirms the potential of the bioconjugation technology to provide simply manufactured and efficacious vaccines against multiple bacterial infections.

## Supplementary Information

ESM 1 (PPTX 212 kb)

ESM 2 (DOCX 60 kb)

## Data Availability

All data has been disclosed.
