# Supplementary material for: The development and characterization of an E. coli O25B bioconjugate vaccine
Source: Glycoconj J. 2021 Mar 17;38(4):421–35. doi: 10.1007/s10719-021-09985-9 (PMC8260533; doi:10.1007/s10719-021-09985-9)
Supplement: Supplementary file 1 — (PPTX 212 kb) [file 10719_2021_9985_MOESM1_ESM.pptx]

## Slide 1
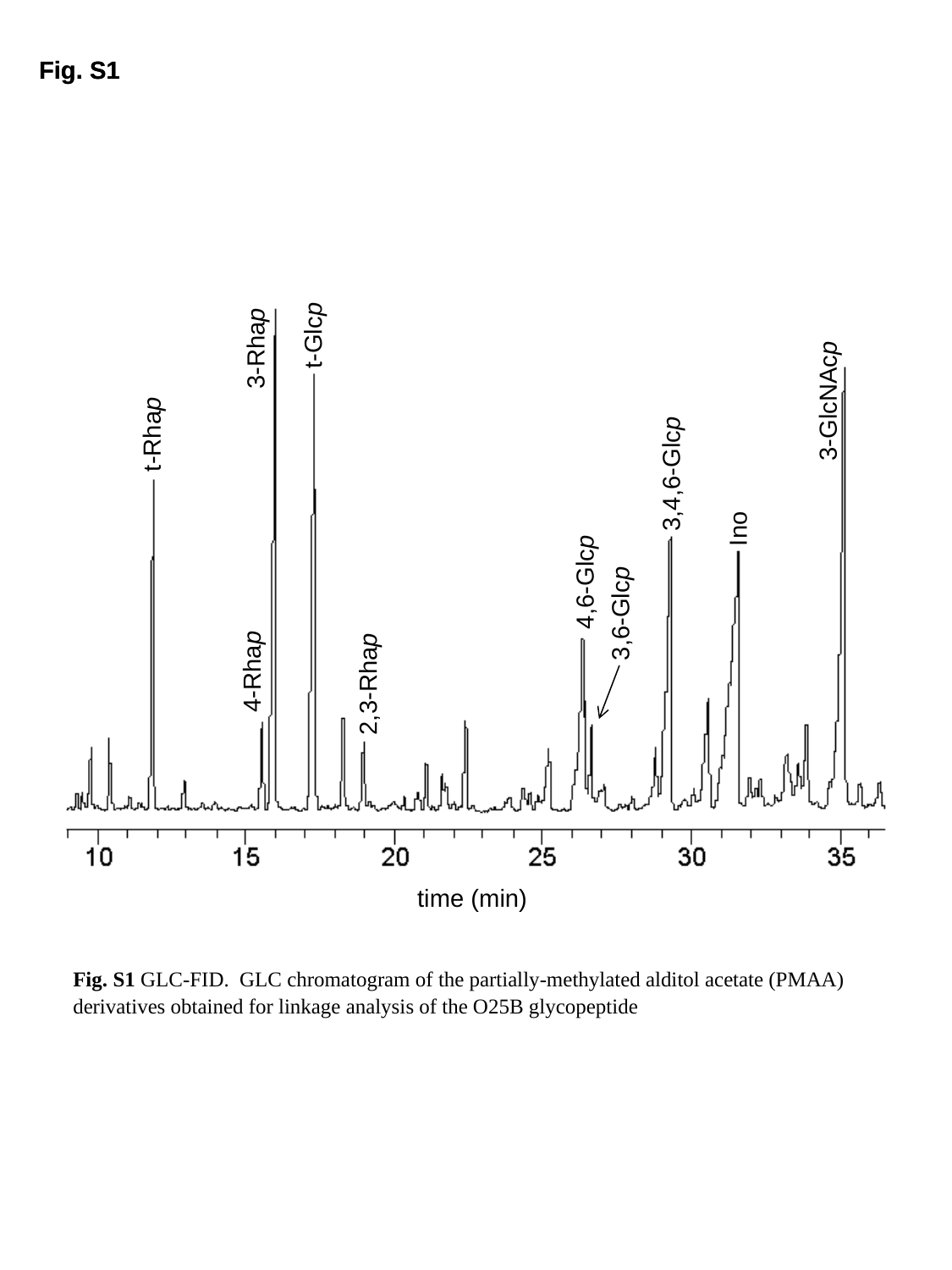

Fig. S1
Fig. S1
t-Glcp
3-Rhap
3-GlcNAcp
t-Rhap
3,4,6-Glcp
Ino
4,6-Glcp
3,6-Glcp
2,3-Rhap
4-Rhap
time (min)
Fig. S1 GLC-FID. GLC chromatogram of the partially-methylated alditol acetate (PMAA) derivatives obtained for linkage analysis of the O25B glycopeptide

## Slide 2
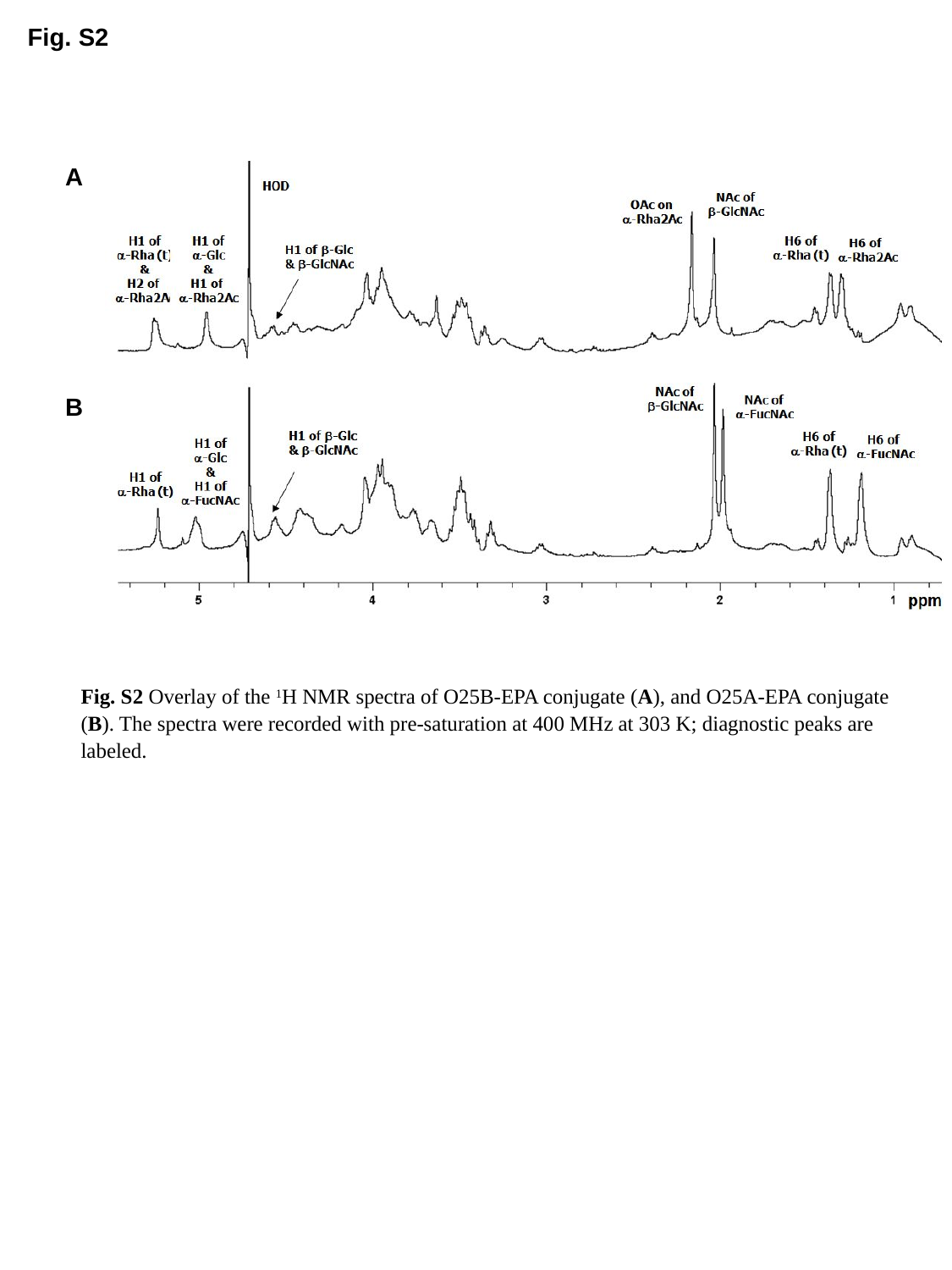

Fig. S2
A
B
Fig. S2 Overlay of the 1H NMR spectra of O25B-EPA conjugate (A), and O25A-EPA conjugate (B). The spectra were recorded with pre-saturation at 400 MHz at 303 K; diagnostic peaks are labeled.

## Slide 3
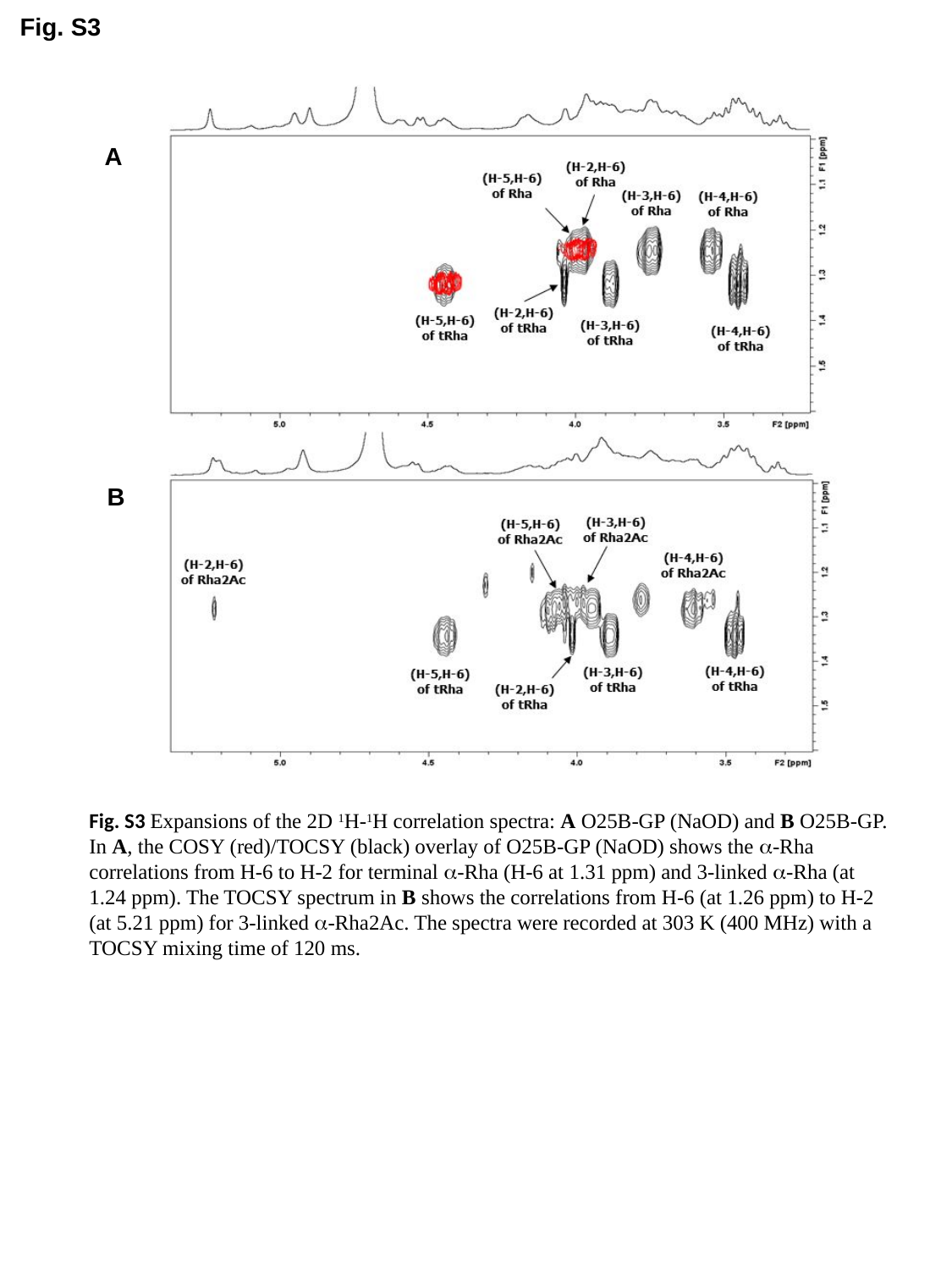

Fig. S3
A
B
Fig. S3 Expansions of the 2D 1H-1H correlation spectra: A O25B-GP (NaOD) and B O25B-GP. In A, the COSY (red)/TOCSY (black) overlay of O25B-GP (NaOD) shows the a-Rha correlations from H-6 to H-2 for terminal a-Rha (H-6 at 1.31 ppm) and 3-linked a-Rha (at 1.24 ppm). The TOCSY spectrum in B shows the correlations from H-6 (at 1.26 ppm) to H-2 (at 5.21 ppm) for 3-linked a-Rha2Ac. The spectra were recorded at 303 K (400 MHz) with a TOCSY mixing time of 120 ms.
