# Supplementary material for: The development and characterization of an E. coli O25B bioconjugate vaccine
Source: Glycoconj J. 2021 Mar 17;38(4):421–35. doi: 10.1007/s10719-021-09985-9 (PMC8260533; doi:10.1007/s10719-021-09985-9)
Supplement: Supplementary file 2 — (DOCX 60 kb) [file 10719_2021_9985_MOESM2_ESM.docx]

**Supplementary Material to:**

**The development and characterization of an *E. coli* O25B bioconjugate vaccine**

Michael Kowarik^1,2*^, Michael Wetter^1, 3^, Micha A. Haeuptle^1, 4^, Martin Braun^1, 2^, Michael Steffen^1, 2^, Stefan Kemmler^1, 5^, Neil Ravenscroft^6^, Gianluigi De Benedetto^7, 8^, Matthias Zuppiger^1, 2^, Dominique Sirena^1, 2, 9^, Paola Cescutti^7^, and Michael Wacker^1, 10^

From ^1^GlycoVaxyn AG, Grabenstrasse 3, 8952 Schlieren, Switzerland, ^2^LimmaTech Biologics AG, Grabenstrasse 3, 8952 Schlieren, Switzerland, ^6^Department of Chemistry, University of Cape Town, Rondebosch 7701, South Africa, ^7^Dip. di Scienze della Vita, University di Trieste, 34127 Trieste Italy

^3^Current address: Institute of Microbiology, ETH Zurich, Vladimir-​Prelog-Weg 1-5/10, 8093 Zürich, ^4^Current Address: Molecular Partners AG, Wagistrasse 14, 8952 Schlieren, Switzerland ^5^Current Address: Numab Therapeutics AG, Einsiedlerstrasse 34, 8820 Wädenswil, Switzerland, , ^8^Current address: National Institute for Biological Standards and Control, Blanche Lane, South Mimms, Potters Bar, Hertfordshire, EN6 3QG, United Kingdom, ^9^Current address: GlycoEra AG, Grabenstrasse 3, 8952 Schlieren, Switzerland , ^10^Current address: Wacker Biotech Consulting AG, Heuelstrasse 22, 8800 Thalwil, Switzerland

Correspondence to Michael Kowarik, LimmaTech Biologics AG, Grabenstrasse 3, 8952 Schlieren, Switzerland;

E-mail: [michael.kowarik@lmtbio.com](mailto:michael.kowarik@lmtbio.com)

**Supplementary Tables**

**Table S1**

| Plasmids | Description | Ref |
| --- | --- | --- |
| p112 | pACT3 based expression plasmid for IPTG inducible PglB containing a C terminal HA tag, SpecR | [1] |
| p114 | pEXT21 based expression plasmid for IPTG inducible PglB containing a C terminal HA tag, SpecR | [1] |
| p150 | pBR322 based expression plasmid for arabinose inducible epa containing a His tag and two glycosylation sites, AmpR | [2] |
| p659 | pBR322 based expression plasmid for arabinose inducible EPA like p150, but containing an additional N and C terminal tag encoding each one glycosylation site, resulting in a total of 4 glycosylation sites and no His-tag, AmpR | This study |
| p939 | pEXT21 based expression plasmid for IPTG inducible PglB containing a C terminal HA tag, PglB-HA DNA was codon usage optimized and synthetized, SpecR | This study |
| p970 | pEXT21 based expression plasmid for IPTG inducible PglB as p114, containing a codon usage optimized pglB, SpecR | [2, 3] |
| p1010 | Synthetized pTKRED (GU327533.1), encoding inducible lambda red recombinase and homing endonuclease SceI genes | [4] |
| p1046 | pDOC-E based donor plasmid for replacement of W3110 *rfb* cluster with the *rfb* cluster of O25B strain upec138 | This study |
| p1076 | p659 with clmR resistance cassette replaced by kanR from pKDx (Datsenko and Wanner, 25) | This study |
| pDOC-E | Donor-plasmid for homologous recombination containing a NotI site flanked by SceI sites for donor cargo DNA cloning, the sacB gene for counterselection, ampR, OriT, pMB1 ori | [5] |
| pACT3 | IPTG inducible expression plasmid, clmR, ori p15A | [6] |
| pEXT21 | IPTG inducible expression plasmid, specR, ori IncW from pSF6 | [6] |
|  |  |  |
| Strains |  |  |
| *E. coli* W3110 | *E. coli* lab strain, obtained from The Coli Genetic Stock Center (http://cgsc.biology.yale.edu) | CGSC# 4474 |
| UPEC138 | O25B clinical isolate | [7] |
| UPEC 177 | O25B clinical isolate | [7] |
| UPEC 350 | O25B clinical isolate | [7] |
| UPEC 436 | O25A clinical isolate | [7] |

**Table S2**

|  | **EcO25B-EPA** | **EcO25A-EPA** |
| --- | --- | --- |
| **Acetate [μg/mL] injection 1** | **6.33** | **< 1.70** |
| **Acetate [μg/mL] injection 2** | **6.56** | **< 1.70** |
| **Acetate [μg/mL] injection 3** | **6.26** | **< 1.70** |
| **Acetate [μg/mL] injection 4** | **6.58** | **< 1.70** |
| **Average acetate [μg/mL]** | **6.43** | **< 1.70** |
| **STDEV [μg/mL]** | **0.16** | **N/A** |
| **CV [%]** | **2.5** | **N/A** |
| **Dilution factor** | **0.9** | **0.9** |
| **Adjusted acetate [μg/mL]** | **7.15** | **< 1.89** |
| **PS concentration [μg/mL]** | **149.3** | **246.3** |
| **Acetate / PS [% m/m]** | **4.8** | **< 0.77** |
| **Degree of O-acetylation [%]** | **98.1** | **< 15.8** |

References

1. Ihssen, J., Kowarik, M., Dilettoso, S., Tanner, C., Wacker, M., Thony-Meyer, L.: Production of glycoprotein vaccines in Escherichia coli. Microb Cell Fact (2010). https://doi.org/10.1186/1475-2859-9-61

2. Wacker, M., Wang, L., Kowarik, M., Dowd, M., Lipowsky, G., Faridmoayer, A., Shields, K., Park, S., Alaimo, C., Kelley, K.A., Braun, M., Quebatte, J., Gambillara, V., Carranza, P., Steffen, M., Lee, J.C.: Prevention of Staphylococcus aureus Infections by Glycoprotein Vaccines Synthesized in Escherichia coli. J Infect Dis (2014). https://doi.org/10.1093/infdis/jit800

3. Ihssen, J., Haas, J., Kowarik, M., Wiesli, L., Wacker, M., Schwede, T., Thony-Meyer, L.: Increased efficiency of Campylobacter jejuni N-oligosaccharyltransferase PglB by structure-guided engineering. Open Biology (2015). https://doi.org/10.1098/rsob.140227

4. Kuhlman, T.E., Cox, E.C.: Site-specific chromosomal integration of large synthetic constructs. Nucleic Acids Res (2010). https://doi.org/10.1093/nar/gkp1193

5. Lee, D.J., Bingle, L.E., Heurlier, K., Pallen, M.J., Penn, C.W., Busby, S.J., Hobman, J.L.: Gene doctoring. a method for recombineering in laboratory and pathogenic Escherichia coli strains. BMC microbiology **9**, 252 (2009)

6. Dykxhoorn, D.M., St Pierre, R., Linn, T.: A set of compatible tac promoter expression vectors. Gene **177**, 133–136 (1996)

7. Kowarik, M., Wetter, M., Kemmler, S., Häuptle, M., Gambillara, V., Mally, M.: Polysaccharide and uses thereof Patent US10441647B2
